# Evaluation of Loquat Jam Quality at Different Cooking Times Based on Physicochemical Parameters, GC-IMS and Intelligent Senses

**DOI:** 10.3390/foods13020340

**Published:** 2024-01-22

**Authors:** Mingfeng Qiao, Huan Xiong, Xuemei Cai, Yuqin Jiang, Xinxin Zhao, Baohe Miao

**Affiliations:** 1 Institute of Urban Agriculture, Chinese Academy of Agricultural Sciences, Chengdu National Agricultural Science & Technology Center, Chengdu 610213, China; mfqiao@163.com (M.Q.); yuqinj07@foxmail.com (Y.J.); 15008313421@163.com (X.Z.); 2 Culinary Science Key Laboratory of Sichuan Province, Sichuan Tourism University, Chengdu 610100, China; xionghuan0629@163.com (H.X.); cxm121517@163.com (X.C.); 3 College of Life Science, Dalian Minzu University, Dalian 116600, China

**Keywords:** loquat jam, cooking time, physicochemical parameters, GC-IMS, intelligent senses

## Abstract

**Highlights:**

**What are the main findings?**

- The color, energy, and texture properties of loquat jam changed with the prolongation of cooking time.
- The changes in the content of free amino acids, organic acids and volatile flavor substances with the cooking time caused variation in the olfactory and gustatory senses of loquat jam.
- There was a significant difference in the E-nose and E-tongue results of loquat jam cooked for 100 min and 120 min.

**What is the implication of the main finding?**

- Heating and the resulting intramolecular interactions caused changes in the physicochemical parameters of loquat jam.
- Free amino acids, organic acids, and volatile substances contributed significantly to the flavor composition of loquat jam.
- When the cooking time reached 120 min, the olfactory and gustatory senses of loquat jam underwent significant changes.

**Abstract:**

The study compared and analyzed the quality of loquat jam with different cooking times through physicochemical parameters, headspace-gas chromatography-ion migration spectroscopy (HS-GC-IMS) and intelligent senses. The results showed that with the prolongation of the cooking time, the color of loquat jam slowly deepened, the energy significantly increased, the adhesiveness, gumminess, hardness and chewiness enhanced, the free amino acid content increased from 22.40 to 65.18 mg/g. The organic acid content increased from 1.64 to 9.82 mg/g. Forty-seven volatile flavor compounds were identified in five types of loquat jam using HS-GC-IMS, among which the relative content of aldehydes was sharply higher than that of other chemical substances, playing an important role in the flavor formation of loquat jam. LJ0, LJ1 and LJ2 had higher aldehyde content, followed by LJ3 and LJ4 had the lowest aldehyde content. The orthogonal partial least squares-discriminant analysis (OPLS-DA) screened 15 marker compounds that could distinguish five types of loquat jam. The E-nose results showed a significant difference in olfactory sense between loquat jam cooked for 100 and 120 min. The E-tongue results corroborated the results of free amino acids (FAAs) and organic acids, indicating that the gustatory sense of loquat jam changed significantly when the cooking time reached 120 min. The results provided a basis for further research on the relationship between the cooking process and quality characteristics of loquat jam.

## 1. Introduction

Loquat (*Eriobotrya japonica* Lindl.) is an evergreen fruit tree of the Rosaceae family, and its fruit is used as a dual-purpose medicine and food that has been cultivated in China for more than 2000 years [1]. As a characteristic fruit of South China, it has the effects of clearing the pharynx, moistening the lungs, alleviating cough and lowering phlegm [2], and these effects could help improve the symptoms of COVID-19. Due to the concentrated maturity period and high water content, loquat fruit senescence is rapid after harvest and prone to rot and browning [3]. Therefore, the fresh fruit processing technology of loquat has gradually become a research focus [4].

At present, research on loquat processing products mainly involves dried fruits [5], jelly [6], probiotic yogurt [7], beer [8] and fermented fruit juice [9]. Loquat jam is also one of the loquat processing products, which could not only solve the problem of loquat storage but also partially retain the pharmacological function of loquat [10]. However, current research on loquat jam mainly focuses on its pharmacological effects, and there has been no systematic study on the differences in physicochemical properties and flavor characteristics of loquat jam cooked at different times [11]. Moreover, there is relatively little research on the nutritional components and flavor substances of loquat jam. The cooking process of loquat jam is a complex process of physicochemical changes, and its quality characteristics are greatly influenced by the cooking time [12]. Loquat pulp contains a large amount of organic matter, which is unstable and prone to a series of biochemical reactions during the cooking process, leading to changes in the color, texture and flavor of loquat jam [13]. Therefore, an appropriate cooking time could improve the sensory quality of loquat jam.

The sensory quality of food mainly includes color, texture and flavor [14]. The colorimeter is an electronic instrument that can measure the surface color of an object, representing any color through L*, a*, b*, C* and h* values [13]. The texture analyzer utilizes mechanical principles to determine multiple parameters of food, such as adhesiveness, hardness, gumminess and chewiness, by applying force and measuring deformation [15]. The flavor characteristics of food are composed of flavor amino acids, organic acids, volatile substances, etc. [16]. Amino acid analyzers, high-performance liquid chromatography (HPLC) and gas chromatography-ion mobility spectrometry (GC-IMS) can be used to qualitatively and quantitatively analyze free amino acids, organic acids and volatile components in food [17,18,19]. E-nose and E-tongue are intelligent sensory analysis technologies developed by simulating human olfactory and gustatory senses [20]. Unlike artificial sensory evaluation, they have the advantages of simple operation and efficient analysis and are not affected by subjective factors [21]. They provide useful methods for analyzing the sensory characteristics of food. In recent years, there have been increasing reports on the use of the aforementioned instruments to study the sensory quality of food [22,23,24]. Calory Answer, also known as a food calorie analyzer, uses the principle of near-infrared spectroscopy to provide a reference for analyzing food energy. It is a newly emerging intelligent analysis instrument with relatively few reports in food research in recent years.

In this study, the physicochemical indexes such as color, energy, texture properties, free amino acid contents and organic acid contents of loquat jams cooked at different times were characterized. The volatile compounds of loquat jams with different cooking times were analyzed by GC-IMS. At the same time, a comprehensive analysis was conducted on the olfactory and gustatory senses of loquat jam at different cooking times using E-nose and E-tongue. The quality characteristics of loquat jam with different cooking times were compared by combining physicochemical indexes, GC-IMS and intelligent senses, which have a certain guiding significance for improving the cooking process and quality control of loquat jam.

## 2. Materials and Methods

### 2.1. Material and Reagents

The “Longquan No.1” loquats with uniform size, consistent appearance and fresh and undamaged appearance used for the experiment were purchased from the local fruit supermarket (Chengdu City, China). The size of the purchased loquat was (5.09 ± 0.23) cm × (4.78 ± 0.19) cm, weighting 59.45 ± 4.01 g, with a soluble solid content of approximately 11.90%. White granulated sugar was purchased from the local market (Chengdu City, China).

Analytical purity: sulfosalicylic acid (Sinopharm Chemical Reagent Co., Ltd., Shanghai, China). Chromatographic purity: ninhydrin (Meryer Biochemical Technology Co., Ltd., Shanghai, China); potassium dihydrogen phosphate, sodium dihydrogen phosphate (Sigma Aldrich Trading Co., Ltd., Shanghai, China); tartaric acid, succinic acid, lactic acid, malic acid, acetic acid, citric acid (Aladdin Biochemical Technology Inc., Shanghai, China); Ultrapure water (18.2 MΩ cm^−1^, Milli-Q Plus system, Millipore, Bedford, MA, USA) was used throughout the work.

### 2.2. Production of Loquat Jam

The loquats were cleaned, drained, blanched in boiling water at 95–100 °C for 30 s, fished out, and then quickly cooled by ultrapure water to below 40 °C. The pedicle, peel and core of the loquats were removed. The cleaned loquat pulp was poured into a homogenizer (L10-L191, Jiuyang Inc., Jinan City, China) and beaten for 30 s. 2 kg of loquat homogenized liquid was weighed and poured into the pot. The slurry and white granulated sugar were mixed evenly at a mass ratio of 5:1, heated and simmered and stirred continuously during the simmering. The cooking time was set to 80 min (called LJ1), 100 min (called LJ2), 120 min (called LJ3) and 140 min (called LJ4), and the uncooked loquat homogenate liquid was set as a blank control group, called LJ0. After the loquat jam was made, it was placed in a clean and dry glass bottle while hot. When the temperature dropped to around 25 °C, it was sealed and stored in a 4 °C refrigerator for analysis.

### 2.3. Determination of Physicochemical Parameters

#### 2.3.1. Colorimetric Analysis

A colorimeter (NR200+, 3 nh Intelligent Technology Co., Ltd., Guangzhou City, China) was used to measure the trichromatic values of loquat jam, including L* (lightness), a* (red-green coordinate) and b* (yellow-blue coordinate). Additionally, C* (chroma) and h* (hue) were measured.

#### 2.3.2. Energy Analysis

The content of energy, carbohydrates, protein, fat, etc., in loquat jam was measured using the Calory Answer (CA-HM, JWP, Tokyo, Japan).

#### 2.3.3. Textural Properties Analysis

Texture profile analysis (TPA) was performed using a texture analyzer (TMS-Pro, FTC, Sacramento, CA, USA) [7]. 250 g of loquat jam was placed into a cylindrical glass bottle with a diameter of 5 cm, refrigerated, and allowed to stand for 24 h before being used for determination. The P2.5 probe was used for testing. The loquat jam sample was compressed in TPA for two cycles (double compression test model). The test speed was 1 mm/s, the triggering force was 0.375 N, the down-pressing distance was 25 mm, the interval was 30 s, and the down-pressing deformation variable was 65%.

#### 2.3.4. Determination of Free Amino Acids

An Automatic Amino Acid Analyzer (S433D, Sykam, Munich, Germany) was used to determine the content of free amino acids [17]. The sample was pretreated using the sulfosalicylic acid hydrolysis method. 20 g of loquat jam was homogenized with ultrapure water in a 1:1 mass ratio. After homogenization, the sample was sonicated (SG900HPT, Xinling Instrument Equipment Co., Ltd., Zhengzhou City, China) with a 7% sulfosalicylic acid solution in a 1:1 mass ratio at 55 KHz for 40 min and then filtered. The filtrate was centrifuged (TGL-18, Shuke Instrument Co., Ltd., Chengdu City, China) at a speed of 10,000 r/min for 15 min, and 1 mL of supernatant was filtered through a 0.22 μm microporous membrane (Sigma Aldrich Trading Co., Ltd., Shanghai, China) into a sample bottle for testing. The free amino acid content was calculated by the instrument’s built-in program, which integrated the sample spectrum that needed to be calculated, and the program would automatically calculate based on the selected calibration file. Analysis conditions: PEEK analysis column (4.6 mm × 150 mm, 7 μm), 10% cross-linking; column temperature 20–99 °C; reactor temperature 130 °C; Detection wavelengths of 570 and 440 nm; analysis time 57 min. The flow rate of ninhydrin reagent was 0.25 mL/min; injection volume 40 μL.

#### 2.3.5. Determination of Organic Acids

HPLC (E2695, Waters, Milford, CT, USA) was used to determine the content of organic acids [25]. An amount of 25 g of loquat jam was added to ultrapure water and diluted into a 50 mL volumetric flask, heated and extracted in a 75 °C water bath for 1 h, cooled to room temperature and then filtered. The filtrate was centrifuged at 16,000× *g* r/min for 20 min, and 1 mL of the supernatant was filtered through a 0.45 μm microporous membrane (Anavo Technology Co., Ltd., Beijing, China) into a sample bottle for testing.

The HPLC conditions were as follows: The instrument was equipped with a 717+ automatic injector with 20 μL once and a 2996 Photodiode Array detector (PDA) UV at 210 nm. Separation was achieved using an Agilent Zorbax SB-C_18_ column (5 μm, 4.6 mm × 250 mm) at 25 °C. The mobile phase was 20 mmol/L NaH_2_PO_4_ (0.01 mol/L potassium dihydrogen phosphate solution was used with a pH of 2.60), with a flow rate of 1.00 mL/min. Each organic acid was quantified by the standard curve of the authentic product. The standard curves for the six measured organic acids are shown in Table 1 below.

### 2.4. Analysis of Volatile Compounds by HS–GC-IMS

The volatile compounds of loquat jam were analyzed by HS–GC-IMS (FlavourSpec, G.A.S., Dortmund, Germany) [26]. A sample of 5 g was placed into a headspace bottle and sealed. The test used an automated headspace sampling method with an injection volume of 100 μL, incubation time of 15 min, incubation temperature of 60 °C, injection needle temperature of 85 °C, and incubation speed of 500 r/min.

Using the MXT-WAX column (15 m × 0.53 mm × 1 μm), GC separation was performed at 40 °C for 30 min. The programmed flow of the carrier gas (nitrogen, 99.999% purity) was set as follows: the initial flow rate was 2 mL/min held for 5 min, then linearly increased to 10 mL/min within 5 min, finally increased to 100 mL/min within 10 min held for 10 min. The IMS system was coupled to GC and maintained at 45 °C. The speed of the drift gas (nitrogen, 99.999% purity) was kept at 150 mL/min.

RIs were calculated using n-alkanes (C4–C9) as external references. The qualitative analysis of volatile compounds was performed by comparing their RIs and drift times with standards in the GC-IMS library Search (G.A.S, Dortmund, Germany). The relative ratio of flavor components was correlated with the peak signal.

### 2.5. Analysis of Intelligent Senses

#### 2.5.1. Analysis of E-Nose

Olfactory sense analysis was performed with a Fox 4000 E-nose (Alpha MOS, Toulouse, France) equipped with an injection system, 18 sensor chambers (LY2/LG, LY2/G, LY2/AA, LY2/Gh, LY2/gCT1, LY2/gCT, T30/1, P10/1, P10/2, P40/1, T70/2, PA/2, P30/1, P40/2, P30/2, T40/2, T40/1, TA/2), a mass flow controller and an acquisition board with a microcontroller [27]. The main pieces of information granted by each sensor are shown in Table 2. The carrier gas was TOC-grade synthetic air (*p* = 5 psi). Prior to detection, each sample (1 g) was placed in a 10 mL airtight glass vial for 5 min at 50 °C (headspace-generation time and temperature). The measurement phase lasted for 120 s, which was long enough for the sensors to reach stable signal values. After each measurement, zero gas (air filtered by active carbon) was pumped into the sample gas path from the other port of the instrument for 180 s (flush time). The signal data from the sensors were collected by the computer once per second during the measurements, and when the measurement process was completed, the acquired data were stored, respectively. The observation of each sample was repeated five times, and three stable sets of data were retained.

#### 2.5.2. Analysis of E-Tongue

Gustatory sense analysis was performed by *α*-ASTREE E-tongue (Alpha MOS, Toulouse, France), which provided seven sensors for sourness (AHS), saltiness (CTS), umami (NMS), sweetness (ANS), bitterness (SCS) and two reference electrodes (PKS and CPS) [28]. A total of 50 g of the sample was added to 200 g of ultrapure water and sonicated at 55 KHz for 25 min before filtration. Then, 80 mL of the filtrate was injected into a 120 mL beaker for testing. The measuring time was set to 120 s for each sample, and the sensors were rinsed for 10 s using ultrapure water to reach stable potential readings before detecting the next sample. Following the suggestion of Li et al. [29], each sample was repeated eight times, and the data of the last five stable sets were selected as the original data for analysis.

### 2.6. Statistical Analysis

All data were analyzed using IBM SPSS Statistics 26.0 (SPSS Inc., Chicago, IL, USA) and one-factor analysis of variance (ANOVA), and *p* < 0.05 was considered statistically significant. The fingerprint and topographic plots were generated by Gallery Plot and Reporter software (G.A.S., Dortmund, Germany). Origin 2022 (OriginLab Corporation, Northampton, MA, USA) was used for radar plot, line chart, histogram and principal component (PCA) analyses. Chemometrics was performed using SIMCA 14.1 (MKS Umetrics, Umea, Sweden).

## 3. Results and Discussion

### 3.1. Physicochemical Properties Analysis

#### 3.1.1. Colorimetric Analysis

**Figure 1:**
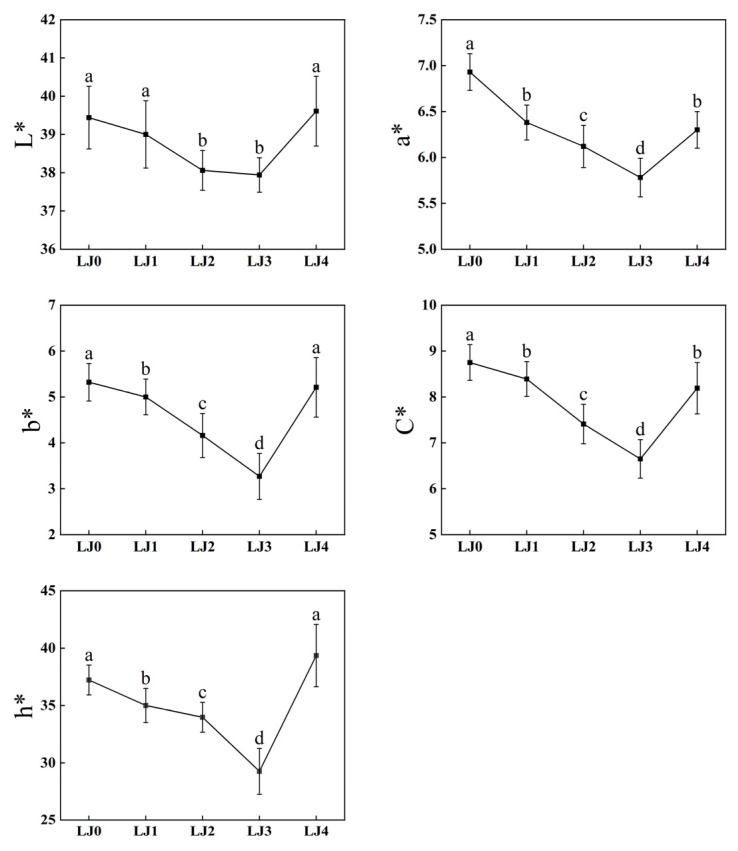
Line chart of colorimetric analysis of loquat jam. Values marked with different letters in the same line chart were significantly different (*p* < 0.05, *n* = 3).

The color of loquat jam with different cooking times was quite different. Therefore, the trichromatic values, chroma and hue of loquat jam were analyzed (Figure 1). The results showed that for LJ0, LJ1, LJ2 and LJ3, their L* (from 39.44 to 37.94), a* (from 6.93 to 5.78), b* (from 5.32 to 3.27), C* (from 8.75 to 6.65) and h* (from 37.22 to 29.26) values generally decreased with the prolongation of cooking time. However, when the cooking time reached 140 min (LJ4), the L* (from 37.94 to 39.61), a* (from 5.78 to 6.31), b* (from 3.27 to 5.21), C* (from 6.65 to 8.19) and h* (from 29.26 to 39.35) values significantly increased (*p* < 0.05). These results indicated that with the prolongation of cooking time, the color of loquat jam changed from bright to dark, from yellow to blue and from red to green, with a decrease in chroma. Nevertheless, when the cooking time reached 140 min, the lightness and chroma of the loquat jam increased, while the red and yellow also increased. The color of loquat jam depends not only on the raw material but also on biochemical reactions, such as caramelization and Maillard reactions during the cooking process [30]. The gradual deepening of the color of loquat jam might be due to the presence of many substrates, which, with the prolongation of reaction time, resulted in the formation of more dark-colored products [31]. As for the sudden increase in the trichromatic value, chroma and hue of loquat jam when the cooking time was increased to 140 min, this might be due to changes in the type and content of colored substances in loquat jam with the extension of the cooking time, resulting in improved color of loquat jam. The specific reasons need further research.

#### 3.1.2. Energy Analysis

**Figure 2:**
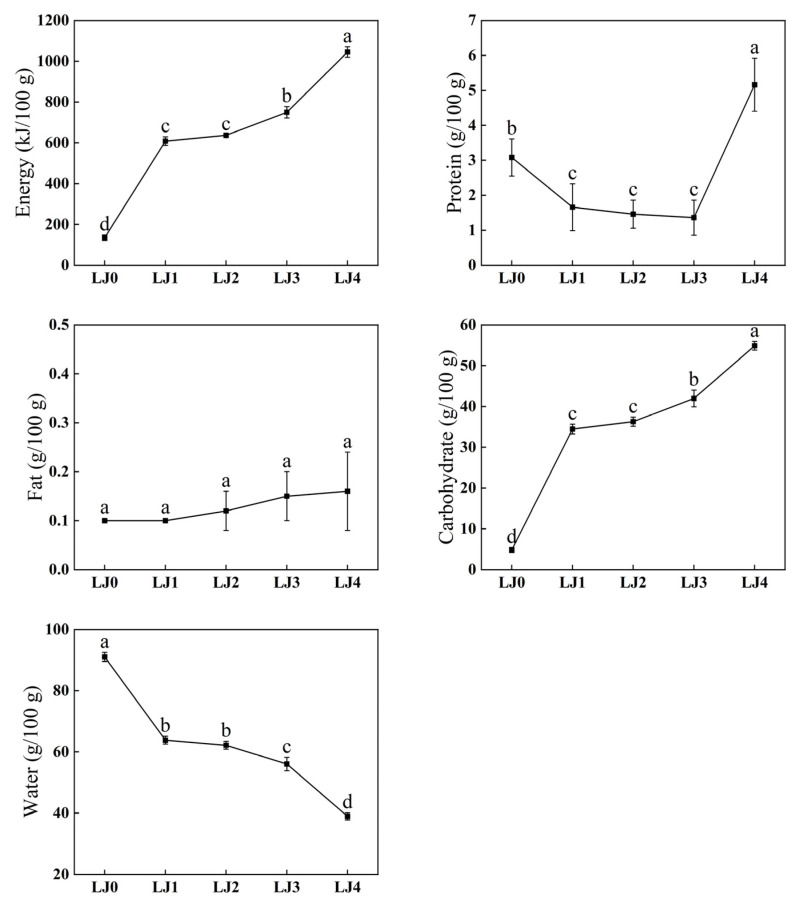
Line chart of energy analysis of loquat jam. Values marked with different letters in the same line chart were significantly different (*p* < 0.05, *n* = 3).

Due to the gradual changes in people’s dietary beliefs and increasing emphasis on the energy of food, the Calorie Answer was used as a reference to detect the energy of loquat jam (Figure 2). The results showed that with the prolongation of cooking time, the energy (from 134.72 to 1045.16 kJ/100 g) and carbohydrate content (from 4.82 to 54.88 g/100 g) of loquat jam significantly increased (*p* < 0.05), while the water content (from 91.02 to 38.90 g/100 g) significantly decreased (*p* < 0.05), and the fat content (from 0.00 to 0.20 g/100 g) was nearly zero without significant change (*p* > 0.05). The protein content first gradually decreased with the extension of the cooking time (from 3.08 to 1.36 g/100 g) but significantly increased when the cooking time reached 140 min (from 1.36 to 5.16 g/100 g) (*p* < 0.05). Due to the evaporation of water by heat, the moisture content of loquat jam gradually decreased with the extension of cooking time. This might also result in a corresponding increase in the content of other substances, such as carbohydrates. At the same time, heating and intermolecular interactions might lead to carbohydrate synthesis reactions and protein decomposition reactions, resulting in changes in the content of carbohydrates and proteins in loquat jam. Since the fat content in the loquat was almost zero and there might be no fat synthesis during cooking, almost no fat was detected in the loquat jam. As for the sudden increase in protein content in LJ4, the reason might be that the degree of decrease in water content during this stage was much greater than the rate of protein loss or the synthesis of new proteins. The reason for this phenomenon needs further study.

#### 3.1.3. Textural Properties Analysis

**Figure 3:**
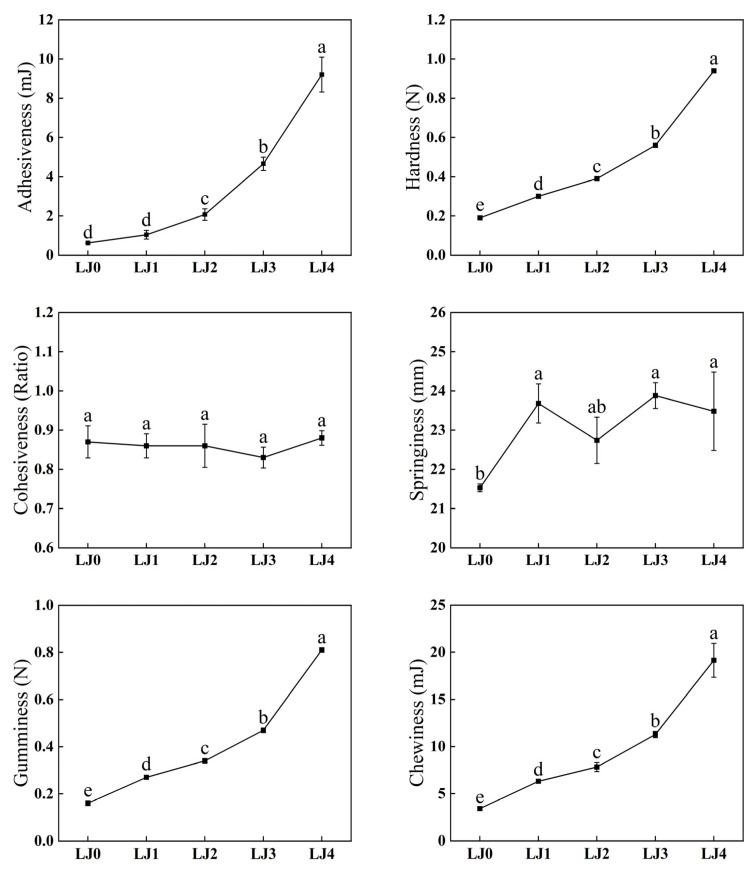
Line chart of textural properties analysis of loquat jam. Values marked with different letters in the same line chart were significantly different (*p* < 0.05, *n* = 3).

The heat transfer and water loss during the cooking process could affect the texture of the loquat jam, so the texture characteristics of the loquat jam were analyzed (Figure 3). The result suggested that as the cooking time prolonged, the adhesiveness (from 0.62 to 9.21 mJ), hardness (from 0.19 to 0.94 N), gumminess (from 0.16 to 0.81 N), and chewiness (from 3.41 to 19.14 mJ) of loquat jam significantly increased (*p* < 0.05). The reason might be that heating caused the water in the loquat jam to evaporate, resulting in intensified intermolecular interactions [32]. However, there was no significant change in the cohesiveness (from 0.83 to 0.88 ratio) of loquat jam with prolonged cooking time, and the springiness (from 22.74 to 23.88 mm) of loquat jam did not significantly change after cooking for 20 min (*p* > 0.05), indicating that cooking for 140 min had little effect on the cohesiveness and springiness of loquat jam.

#### 3.1.4. Free Amino Acids Analysis

**Figure 4:**
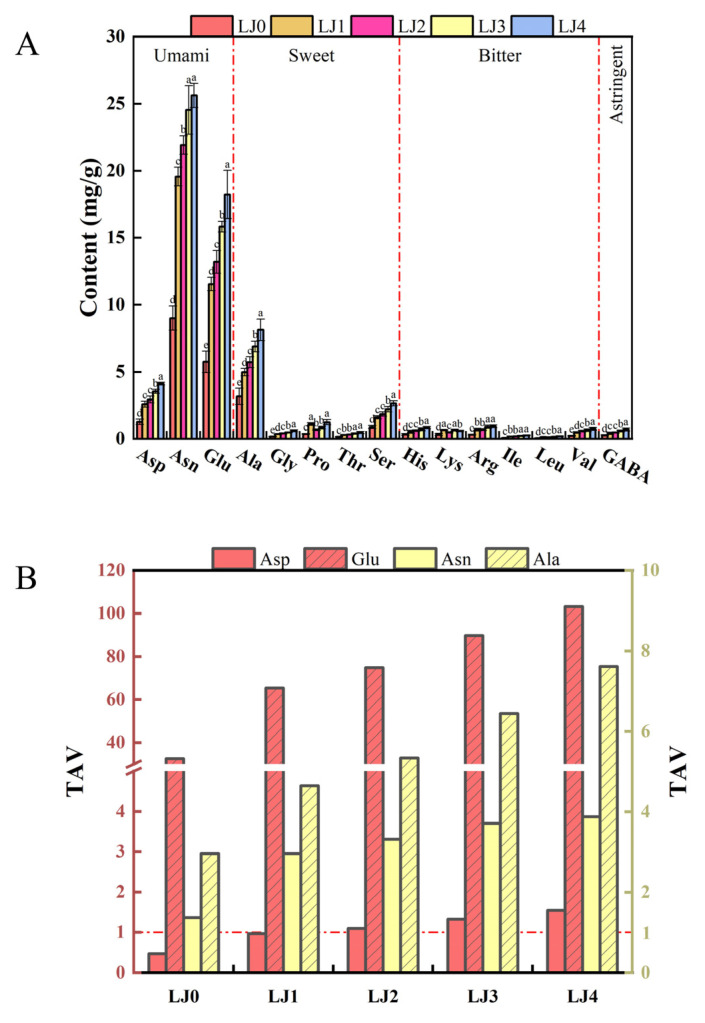
Analysis of free amino acids of the loquat jam. (**A**) Free amino acids content. (**B**) TAVs of free amino acids. The TAVs of free amino acids were calculated by the ratio of their content to the corresponding taste threshold. Values marked with different letters in the five columns of different colors corresponding to the same amino acid of Figure (**A**) were significantly different (*p* < 0.05, *n* = 3).

Free amino acids and organic acids are precursors of aromatic compounds and contributors to taste and nutritional profiles [33]. This work analyzed the contents of free amino acids and organic acids in loquat jam with different cooking times. As shown in Figure 4A, the FAAs contents of loquat jam varied with different cooking times. As the cooking time prolonged, the release content of FAAs significantly increased (*p* < 0.05), with the content of FAAs in the order of LJ0 (22.40 mg/g) < LJ1 (45.01 mg/g) < LJ2 (50.09 mg/g) < LJ3 (58.50 mg/g) < LJ4 (65.18 mg/g), which might be caused by peptides and/or proteins decomposition during the heating process [34]. Free amino acids could be divided into umami amino acids (including Asp, Asn and Glu) (from 16.06 to 47.93 mg/g), sweet amino acids (including Ala, Gly, Pro, Thr and Ser) (from 4.74 to 13.07 mg/g), bitter amino acids (His, Lys, Arg, Ile, Leu and Val) (from 1.33 to 3.49 mg/g), and astringent amino acids (including GABA) (from 0.27 to 0.69 mg/g). Similarly, as the cooking time was prolonged, the content of these four free amino acids also significantly increased (*p* < 0.05). The amounts of these four amino acids accounted for 71.71%, 21.18%, 5.92% and 1.19% of the total FAAs in LJ0, respectively, and those for LJ1 were 74.83%, 18.45%, 5.79% and 0.92%, respectively, and those for LJ2 were 75.93%, 17.88%, 5.24% and 0.95%, respectively, and those for LJ3 were 75.03%, 18.45%, 5.53% and 0.99%, respectively, and those for LJ4 were 73.54%, 20.05%, 5.36% and 1.05%, respectively. With the prolongation of cooking time, the different degrees of participation of these four free amino acids in the Maillard reaction may be the reason for the change in their distribution [35]. To evaluate the contribution of FAAs to the taste of loquat jam, taste activity values (TAVs) of FAAs were calculated (Figure 4B). The TAVs of Asn, Glu and Ala in the five kinds of loquat jam were above 1.0, probably contributing to the umami and sweet taste of loquat jam. The TAVs of Asp in LJ2, LJ3 and LJ4 were also greater than 1.0 and gradually increased with the prolongation of cooking time, which might imply their differences in taste.

#### 3.1.5. Organic Acids Analysis

**Figure 5:**
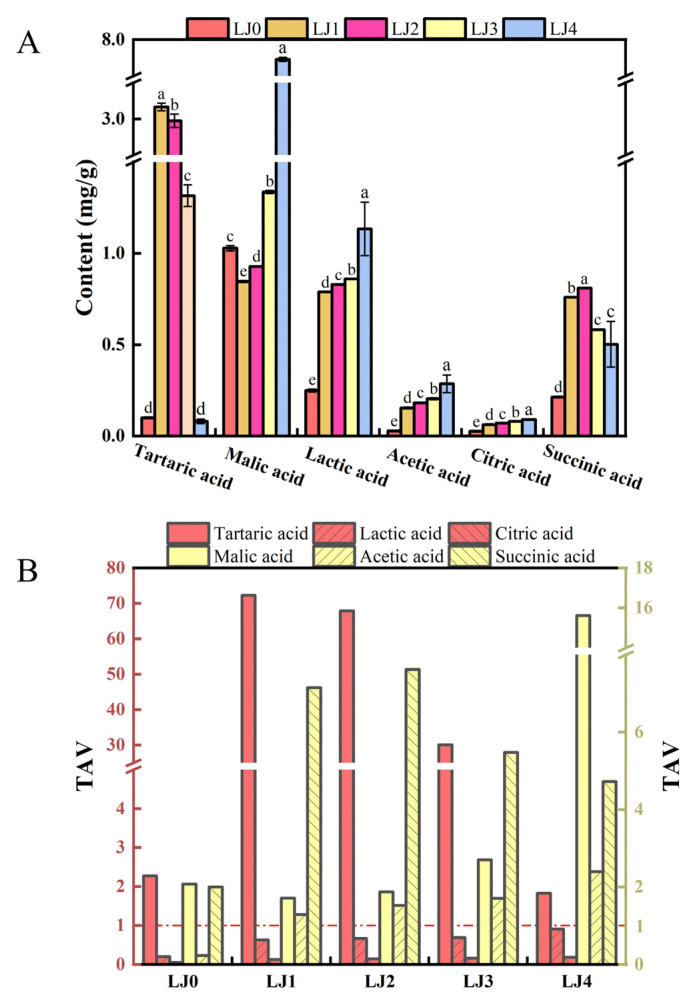
Analysis of organic acids of the loquat jam. (**A**) Organic acids content. (**B**) TAVs of organic acids. The TAVs of organic acids were calculated by the ratio of their content to the corresponding taste threshold. Values marked with different letters in the five columns of different colors corresponding to the same organic acid of Figure (**A**) were significantly different (*p* < 0.05, *n* = 3).

As shown in Figure 5A, loquat jams cooked at different times had the same types of organic acids but different contents. The content of organic acids varied with the prolongation of cooking time. The organic acid content was highest in LJ4 (9.82 mg/g), followed by LJ2 (5.79 mg/g), LJ1 (5.78 mg/g) and LJ3 (4.38 mg/g), with the lowest content in LJ0 (1.64 mg/g). To evaluate the contribution of organic acids to the taste of loquat jam, TAVs of organic acids were calculated. As shown in Figure 5B, the TAVs of tartaric acid, malic acid, and succinic acid in the five loquat jams were all greater than 1.0. Except for LJ0, the TAVs of acetic acid in the other four loquat jam were also greater than 1.0. The TAVs of lactic acid and citric acid in the five loquat jams were all less than 1.0. The results indicated a potentially considerable contribution of tartaric acid, malic acid, acetic acid and succinic acid to the taste of loquat jam. Tartaric acid could contribute a fresh acid taste to loquat jam [22]. Malic acid and acetic acid could provide a mellow taste to loquat jam as two sourness sources. Succinic acid could contribute to the sour, astringent and umami tastes of the loquat jam [31]. These organic acids could coordinate the sugar–acid ratio of loquat jam, and their synergistic effects could make the taste of loquat jam richer.

### 3.2. GC-IMS Analysis

#### 3.2.1. Volatile Compounds Analysis

**Figure 6:**
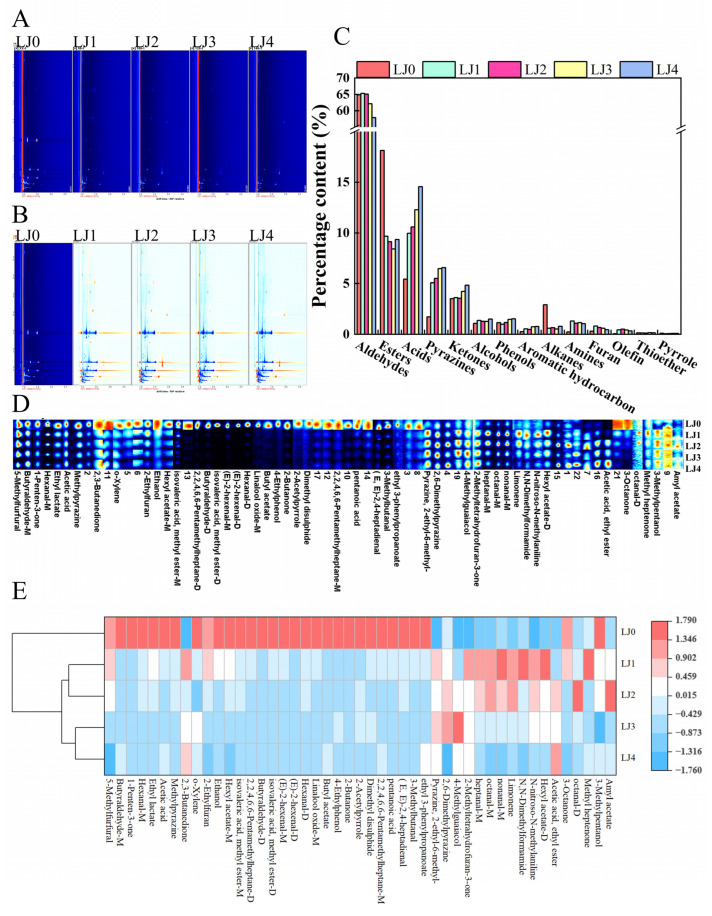
Analysis of volatile compounds of the loquat jam. (**A**) Topographic plots. The dots distributed on both sides of the resistive ionic polymer (RIP) peak represent volatile compounds, and their colors are related to the concentration of volatile compounds. White indicates low concentration; red indicates high concentration; the darker the color, the higher the concentration. (**B**) Differential comparison topographic plots. The concentrations of compounds in LJ1, LJ2, LJ3 and LJ4 are similar to those in LJ0, marked in white. The concentrations higher or lower than LJ0 are marked in red and blue, respectively. The deeper the red/blue color, the higher/lower the concentration of compounds. (**C**) Percentage content of various volatile compounds. (**D**) Fingerprint plots of volatile compounds. (**E**) Cluster heat map of volatile compound concentrations.

HS-GC-IMS was used to analyze the volatile components of loquat jam with different cooking times. As shown in Figure 6A, most signals appeared at the drift time of 1.0–1.8 and the retention time of 200–800 s. A total of 47 volatile flavor compounds were detected and identified in this work (containing monomers and dimers), including 13 aldehydes, 9 esters, 6 ketones, 3 alcohols, 2 acids, 2 phenols, 3 pyrazines, 1 furan, 1 pyrrole, 1 thioether, 1 olefin, 2 alkanes, 2 amines and 1 aromatic hydrocarbon (Table 3).

To better illustrate the differences between loquat jam with different cooking times, the crest intensities of heterogeneous odor compounds on the fingerprint spectrum (Figure 6D) were standardized to grab the approximate percentage of odorant types in loquat jam with different cooking times (Figure 6C). The results showed that the volatile flavor compounds of the five loquat jams were mainly composed of aldehydes, esters and acids, accounting for 57.93–65.35%, 8.40–18.13% and 5.43–14.56% respectively. However, the proportion of olefin, thioether and pyrrole was relatively low, accounting for 0.06–0.52%, 0.12–0.16% and 0.07–0.11%, respectively. The relative content of aldehydes was highest in LJ1, esters were the highest in LJ0 and acids were highest in LJ4. Aldehydes exhibit a grassy and fruity odor at low concentrations. The edge concentration of ketones and alcohols is higher than that of aldehydes, with floral and mellow aromas [36]. Esters are principal products of the esterification reactions among alcohol and acid compounds, modifying the overall flavor of loquat jam. Due to the highest percentage and identified quantity of aldehydes in loquat jam, it was speculated that aldehydes had a prominent contribution to the flavor formation of loquat jam, while thioether and pyrrole had a relatively small contribution to the flavor formation of loquat jam.

From the heat map of volatile compound concentrations (Figure 6E), the relative percentages of characteristic flavor substances in LJ0, such as ethanol, hexyl acetate, methyl isovalerate, butyraldehyde, hexanal, etc., were relatively higher. The concentrations of characteristic flavor compounds in LJ1, such as nonanal, N, N-dimethylformamide, hexyl acetate, methyl heptenone, heptanal, etc., were relatively higher. The percentages of characteristic flavor compounds in LJ2, such as nonanal, amyl acetate, octanal, limonene, etc., were relatively higher. The concentrations of 4-methylguaiacol, 2,6-dimethylpyrazine, etc. were relatively higher in LJ3. Ethyl acetate and 2,3-butanedione had a relatively higher content in LJ4. The types and concentrations of volatile compounds in five types of loquat jam varied with different colors of dots distributed on the sides of the RIP peak (reaction ion peak at drift time 1.0). The differential comparison topographic plots were obtained using the topographic plot of LJ0 as a reference (Figure 6B). Compared with LJ0, the concentrations of various compounds in LJ1, LJ2, LJ3 and LJ4 obviously increased or decreased. In addition, as the cooking time increased, the concentrations of some compounds showed a continuous upward or downward trend, which could be visually reflected in the volatile compound concentration heat map. For example, hexyl acetate-M with pleasant fruit, apple, cherry, pear and floral flavors, 2-ethylfuran with burnt and coffee flavors and hexanal-M with fruity, fatty, green and grassy flavors gradually decreased in concentration with the extension of cooking time, while ethyl acetate with pleasant, sweet and fruity flavors increased in concentration with the prolongation of cooking time. This finding suggested that the volatile profile of the loquat jam changed with the altered cooking time, which could affect the aroma quality of the loquat jam.

GC-IMS and gas chromatography-mass spectrometry (GC-MS) for characterizing volatile flavor compounds in foods could complement each other [36]. Hence, in future research, we will use GC-MS to analyze the chief volatile flavor chemicals in loquat jam with different cooking times to obtain more comprehensive information.

#### 3.2.2. Principal Component Analysis and Orthogonal Partial Least Squares-Discriminant Analysis with Cross-Validation

**Figure 7:**
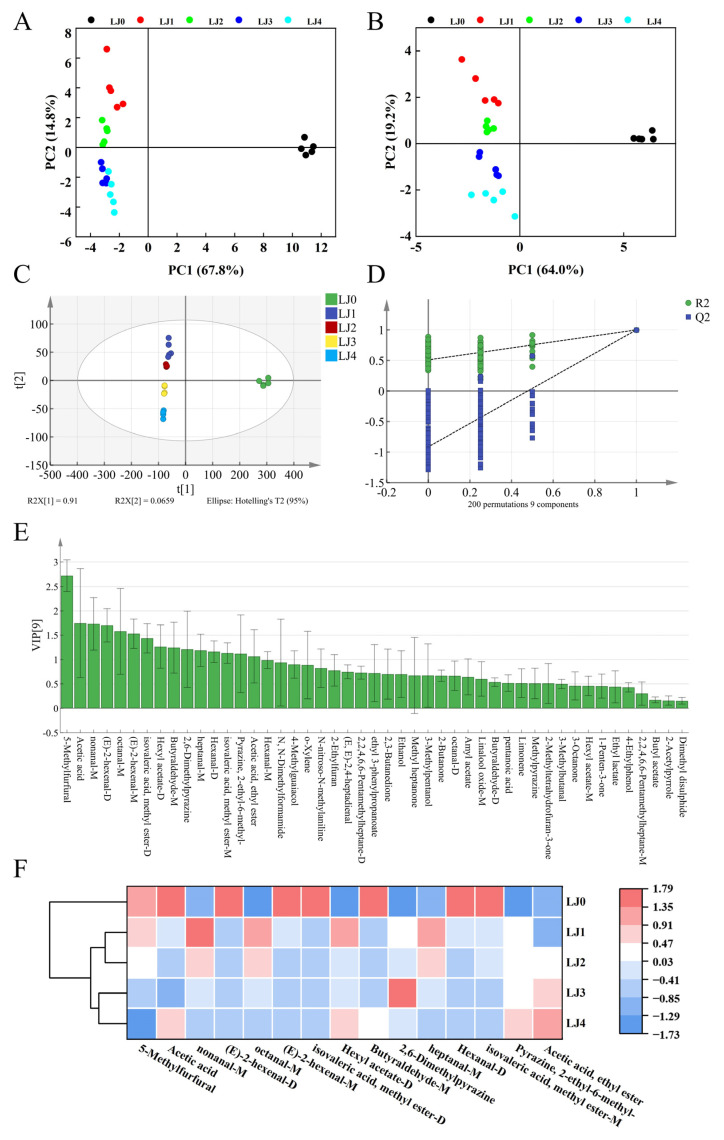
(**A**) PCA scatter map of 47 volatile substances. (**B**) PCA scatter map of 15 differential volatile substances. OPLS-DA scatter plot (**C**) and trans-verifications by a rearrangement trial (**D**) of odor profiles in five loquat jams. The distribution of variable importance in the projection (VIP) (**E**) and clustering heatmap (**F**) of the differential volatile compounds screened from five loquat jam samples.

The GC-IMS quantitative details of five loquat jam samples were assayed by the principal component analysis. As shown in Figure 7A, LJ0 was further away from LJ1, LJ2, LJ3 and LJ4 on PC1. LJ1 and LJ2 were closer to each other on PC2, as were LJ3 and LJ4, but LJ1 and LJ4 were further away from each other on PC2. The same trend could also be observed in the clustering heatmap (Figure 6E), where the five loquat jam varieties were clustered into three groups. The volatile spectrum of LJ0 was significantly different from that of the other four loquat jams. However, LJ1 and LJ2 had a high similarity in their volatile spectra and were classified together, as were LJ3 and LJ4. Therefore, PCA and cluster similarity analysis can be used to distinguish the odor characteristics of the five loquat jam varieties.

OPLS-DA is a useful chemometric calculation approach that could build the interplay simulation among changeful indicators and specimen sorting [37]. *R*^2^*X* and *R*^2^*Y* stand for the explanation rate of the simulation to *X* and *Y* vectors individually, and *Q*^2^ denotes the prediction ability in the actual simulation. The parameters of *R*^2^ and *Q*^2^ higher than 0.5 and near 1.0 were deemed as exact outcomes [38,39]. In the present model (Figure 7C), most information on the volatile components in the five loquat jams was covered by the model with predictive ability *Q*^2^ (cum) = 0.872, integrity-of-simulation indicator *R*^2^*X* (cum) = 0.997 and justifying capacity *R*^2^*Y* (cum) = 0.97. It showed that most loquat jam samples might be classified through the OPLS-DA spread map (Figure 7C), and the categorizing response was close or comparable to the PCA map (Figure 7A). To prevent overestimation, the accuracy of the OPLS-DA model was confirmed via a rearrangement trial and the outcomes are presented in Figure 7D. Followed by 200 trans-verifications, the throwback stripe of simulation *Q*^2^ strides the horizontal ordinate and the nodal increment says minus. All rearrangement trials *R*^2^ and *Q*^2^ are lesser than the raw data, implying that the simulation equation was not over-adapting [40,41]. Therefore, these results obtained by the established OPLS-DA simulation were consistent and sound.

After detecting 47 odorant compounds in five loquat jam samples using GC-IMS, the VIP values obtained through the establishment of a reliable OPLS-DA matching (Figure 7E) were used to estimate the participation of each odorant compound in the loquat jam. The flavor components with VIP values above 1.0 were picked out as the discriminative indicator chemicals in many food specimens [39,42]. The present outcomes found that 15 odor chemicals sieved from the total of odor profiles in five loquat jams (VIP value > 1.0), namely 5-methylfurfural, acetic acid, nonanal-M, (E)-2-hexenal (monomer and dimer), octanal-M, methyl isovalerate (monomer and dimer), hexyl acetate-D, butyraldehyde-M, 2,6-dimethylpyrazine, heptanal-M, hexanal-D, 2-ethyl-6-methyl-pyrazine, ethyl acetate. The PCA and heatmap gathering were performed using these discriminative indicator chemicals (Figure 7B,F). The independence between the five loquat jam samples in Figure 7B was better than that in Figure 7A and most of the specificity of the loquat jam samples could be separated through PCA. The clustering heatmap results indicated that these 15 odor chemicals could better demonstrate the differences among the five loquat jam varieties. The relevant research results suggested that the differential marker odor components sieved by the OPLS-DA coupled with a VIP value above 1.0 could be used for classification between various samples [43,44]. Therefore, screening the differential volatile flavor compounds from loquat jam is feasible based on the OPLS-DA model and VIP values. In the meantime, the results of this study also confirmed that the combination of 15 indicator odor chemicals with PCA and gathering map (Figure 7B,F) could better distinguish the variations in the five loquat jams.

### 3.3. Intelligent Senses Analysis

#### 3.3.1. E-Nose Analysis

**Figure 8:**
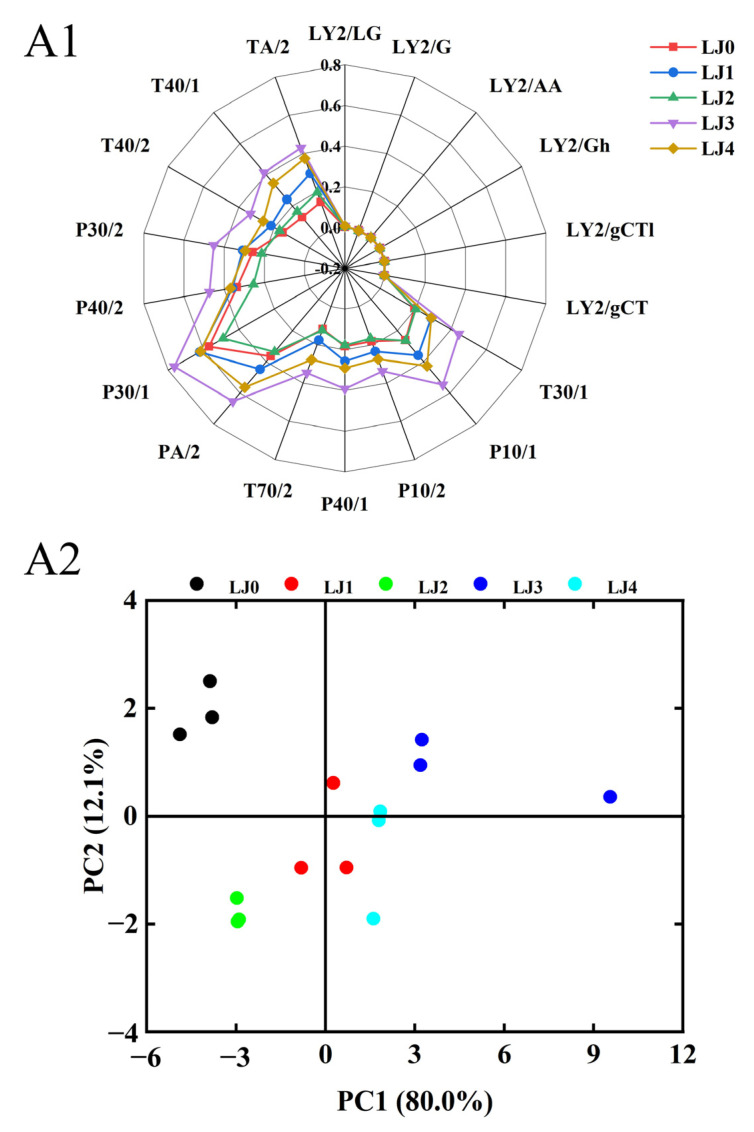
E-nose analysis of the loquat jam. (**A1**) Radar graph for the E-nose analysis. (**A2**) PCA graph for the E-nose analysis.

As shown in Figure 8A1, the response values of the five types of loquat jam on LY2/LG, LY2/G, LY2/AA, LY2/Gh, LY2/gCTI and LY2/gCT sensors were almost zero, possibly due to the low content of sulfur compounds, amines and alkanes in the loquat jam, which was consistent with the results of chromatographic analysis. From the figure, there was a significant difference in the response values of LJ2 and LJ3 on the E-nose sensor. This phenomenon could also be seen in the PCA results (Figure 8A2), where LJ2 was distributed in the third quadrant and LJ3 was distributed in the second quadrant, both of which had significant differences in PC1 and PC2, indicating that there were significant differences in the olfactory sense of these two loquat jams. Meanwhile, the distance between LJ0 and the other four loquat jams in the PCA graph was relatively far, indicating a significant change in the olfactory sense of loquats after cooking.

#### 3.3.2. E-Tongue Analysis

**Figure 9:**
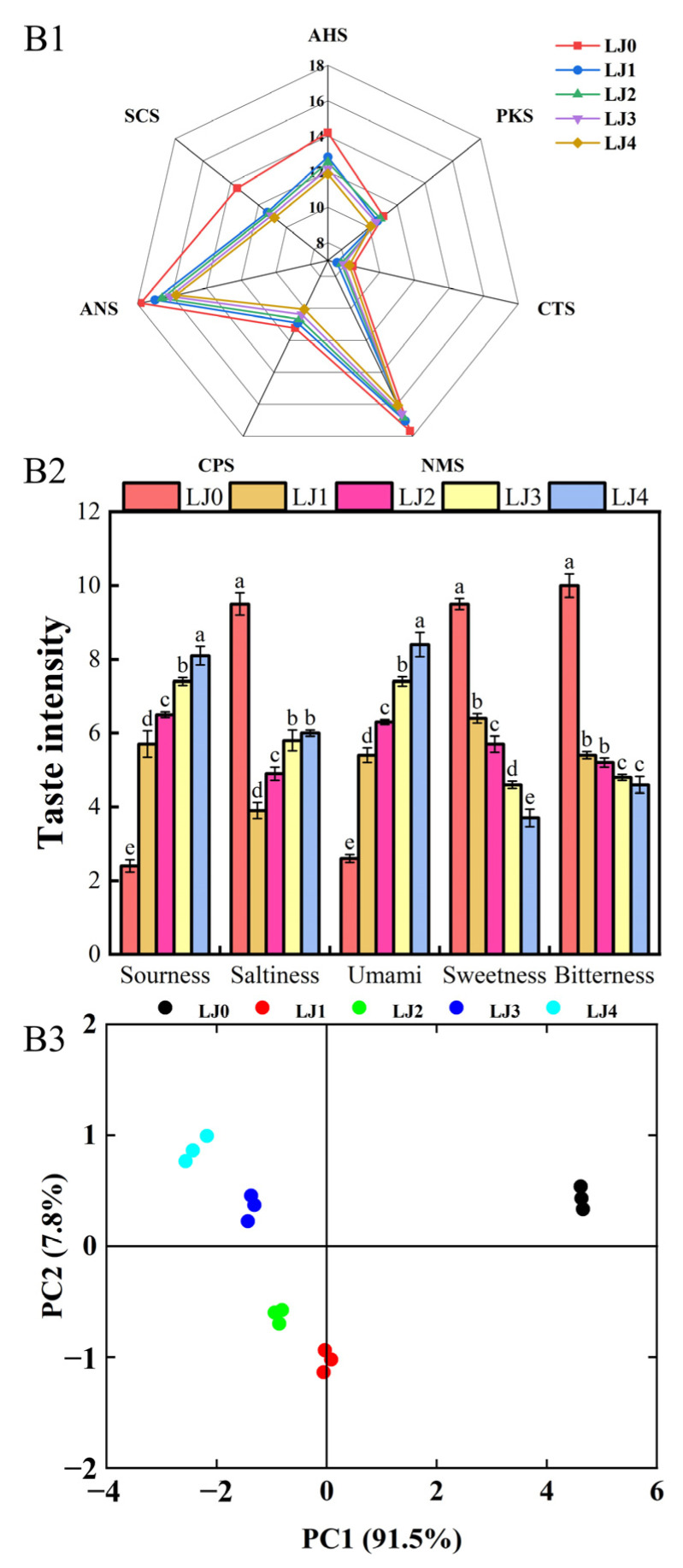
E-tongue analysis of the loquat jam. (**B1**) Radar graph for the E-tongue analysis. (**B2**) Taste intensity histogram. (**B3**) PCA graph for the E-tongue analysis. Values marked with different letters in the five columns of different colors corresponding to the same gustatory sense of Figure (**B3**) were significantly different (*p* < 0.05, *n* = 3).

As shown in Figure 9B1, the response values of loquat jam to various E-tongue sensors changed with the extension of cooking time. The response values of LJ0 and the other four loquat jams on the SCS and AHS sensors showed the largest difference. As the cooking time increased, the umami and sourness of loquat jam gradually increased, while the sweetness and bitterness gradually decreased (Figure 9B2). Moreover, there was a significant difference in the four taste intensities between LJ0 and the other four loquat jams. Compared to LJ0, the saltiness intensity of LJ1 sharply decreased, although it slowly increased with the extension of cooking time but still remained lower than LJ0. These results reflected the differences in gustatory sense between LJ0 and the other four loquat jams. This phenomenon could also be seen in the PCA results (Figure 9B3), where LJ1, LJ2, LJ3 and LJ4 were relatively independent and far away from LJ0. This indicated that as the cooking time prolonged, the taste intensity of loquat jam gradually changed and LJ0 was significantly different from the other four types of loquat jam. LJ1 and LJ2 had a significant difference compared to LJ3 and LJ4 on PC1, indicating a significant change in the gustatory sense of loquat jam when the cooking time reached 120 min.

## 4. Correlation Analysis

### 4.1. Correlation between E-Nose and GC–IMS

**Figure 10:**
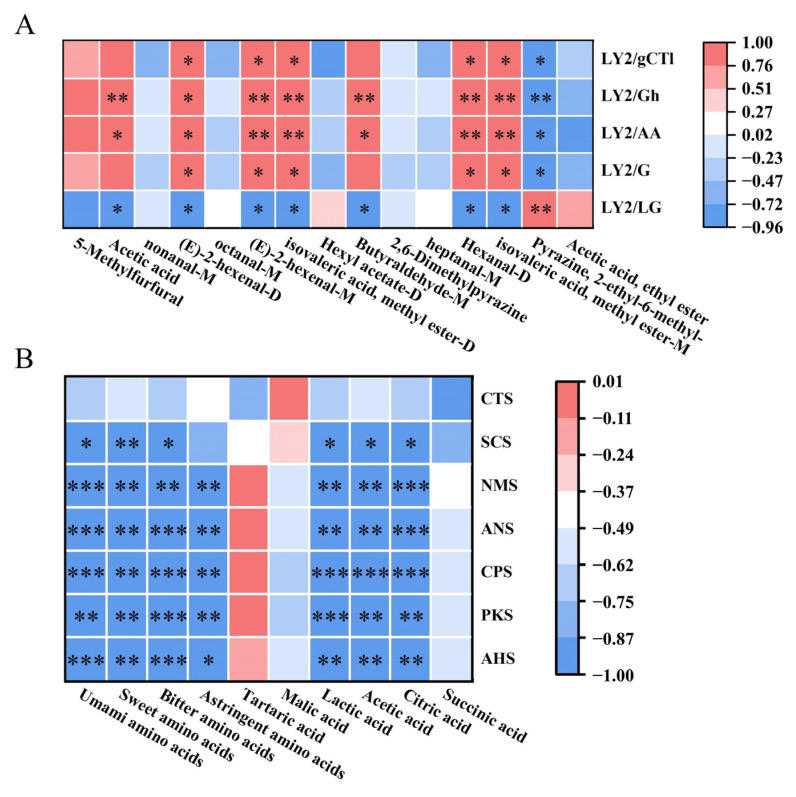
(**A**) The heat map of the correlation between the response values of the E-nose sensors and the levels of differential volatile compounds. (**B**) The heat map of the correlation between the response values of the E-tongue sensors and the contents of FAAs and organic acids. Asterisks *, ** and *** indicate significance at *p* < 0.05, *p* < 0.01 and *p* < 0.001, respectively.

E-nose and GC–IMS can classify five loquat jam samples from different perspectives. For example, the E-nose can provide general information about the volatile compounds in each sample. Conversely, GC–IMS can provide specific volatile characteristics of each loquat jam [21]. Therefore, the potential correlation between the response values of the E-nose sensor and the levels of 15 differential volatile compounds with VIP value > 1.0 detected by GC–IMS was analyzed. As shown in Figure 10A, acetic acid, butyraldehyde-M, LY2/AA and LY2/Gh sensors were positively correlated, while the LY2/LG sensor was negatively correlated. (E)-2-hexenal (monomer and dimer), methyl isovalerate (monomer and dimer) and hexanal-D were positively correlated with LY2/G, LY2/AA, LY2/Gh and LY2/gCT1 sensors, while negatively correlated with LY2/LG sensor. GC-IMS detection results showed that these compounds were highly expressed in LJ0. On the contrary, 2-ethyl-6-methyl-pyrazine was negatively correlated with LY2/G, LY2/AA, LY2/Gh and LY2/gCT1 sensors and positively correlated with LY2/LG sensor. GC-IMS detection results showed that this compound was highly expressed in LJ4. The remaining volatile compounds that did not show significant correlation with the electronic nose sensor, such as nonanal-M, octanal-M and 2,6-dimethylpyrazine, were expressed at higher levels in LJ1, LJ2 and LJ3, respectively. Therefore, the combination of E-nose and GC-IMS can distinguish the five loquat jams based on their olfactory senses.

### 4.2. Correlation between E-Tongue and the Content of FAAs and Organic Acids

The E-tongue can provide information on five taste intensities of loquat jam: saltiness (CTS), umami (NMS), sourness (AHS), sweetness (ANS) and bitterness (SCS). The flavor of amino acids and organic acids contained in loquat jam can affect its taste. Therefore, correlation analysis was conducted between the response values of the E-tongue sensor and the detected content of FAAs of four flavor types and six organic acids. As shown in Figure 10B, four FAAs, lactic acid, acetic acid and citric acid, were negatively correlated with NMS, ANS, CPS, PKS and AHS. Except for astringent amino acids, tartaric acid, malic acid and succinic acid, the remaining three FAAs and organic acids were negatively correlated with SCS. The results indicated that there was a certain correlation between the content of flavor amino acids and organic acids in loquat jam and the response value of E-tongue sensors, which could be used in combination to analyze the taste characteristics of loquat jam.

## 5. Conclusions

In this study, the physicochemical properties, volatile substances and sensory properties of loquat jam with different cooking times were first compared by physicochemical parameters, GC-IMS and intelligent sensory technology. As the cooking time prolonged, the appearance color of the loquat jam slowly deepened, the energy increased obviously, the adhesiveness, gumminess, hardness and chewiness enhanced significantly and the content of free amino acids and organic acids gradually increased. A total of 47 flavor substances were detected and identified from five types of loquat jam using GC-IMS, among which aldehydes, esters and acids were the main chemical components. Due to the highest percentage and identified quantity of aldehydes in loquat jam, it was speculated that aldehydes had a prominent contribution to the flavor formation of loquat jam. The OPLS-DA model analysis screened 15 marker compounds. Combining E-nose with GC-IMS and E-tongue with free amino acids and organic acids can distinguish the five types of loquat jam in terms of olfactory and gustatory senses. When the cooking time reached 120 min, there were significant changes in the olfactory and gustatory senses of loquat jam, indicating that 120 min was a critical time node for the cooking of loquat jam. These results could provide a reference for the production of loquat jam with special physicochemical properties or flavor characteristics and also provide a theoretical basis for the industrial production of loquat jam. In future studies, GC-MS can be used to determine the types and contents of volatile substances in loquat jam with different cooking times to supplement the GC-IMS data and further explore the flavor differences of loquat jam with different cooking times. In addition, professionally trained sensory evaluators can be invited to comprehensively rank the sensory quality of loquat jam with different cooking times and select the most satisfactory cooking time for production guidance.

## Figures and Tables

**Table 1:** The organic acid standard curve.

Name	Retention Time/min	Standard Curves	R^2^
tartaric acid	3.107	y = 1.911x + 7.331	0.9998
malic acid	3.716	y = 0.755x − 0.626	0.9977
lactic acid	4.587	y = 2.706x + 0.999	0.9999
acetic acid	4.846	y = 1.786x − 4.665	0.9999
citric acid	6.601	y = 0.541x + 0.999	0.9998
succinic acid	7.207	y = 0.616x − 0.686	0.9962

**Table 2:** Performance description of the E-nose sensors.

Sensors	Performance Description	Sensors	Performance Description
LY2/LG	Sensitive to oxidizing gas	P40/1	Sensitive to fluorine
LY2/G	Sensitive to ammonia, carbon monoxide	T70/2	Sensitive to aromatic compounds
LY2/AA	Sensitive to ethanol	PA/2	Sensitive to ethanol, ammonia/organic amines
LY2/Gh	Sensitive to ammonia/organic amines	P30/1	Sensitive to polar compounds (ethanol)
LY2/gCT1	Sensitive to hydrogen sulfide	P40/2	Sensitive to heteroatom/chloride/aldehydes
LY2/gCT	Sensitive to propane/butane	P30/2	Sensitive to alcohol
T30/1	Sensitive to organic solvents	T40/2	Sensitive to aldehydes
P10/1	Sensitive to hydrocarbons	T40/1	Sensitive to chlorinated compounds
P10/2	Sensitive to methane	TA/2	Sensitive to air quality

**Table 3:** Volatile compounds detected during five different cooking times of loquat jam.

No	Component Name	CAS	RI	Rt [sec]	Relative Amount/%					Odor Description
					LJ0	LJ1	LJ2	LJ3	LJ4	
1	(E)-2-hexenal-D	C6728263	1223.4	779.477	14.62 ± 0.88 ^a^	2.12 ± 0.16 ^b^	2.19 ± 0.19 ^b^	2.49 ± 0.10 ^b^	2.72 ± 0.32 ^b^	green, fruit
2	(E)-2-hexenal-M	C6728263	1222.1	775.097	14.42 ± 0.31 ^a^	4.75 ± 0.96 ^b^	4.07 ± 0.15 ^b^	4.25 ± 0.30 ^b^	3.99 ± 0.23 ^b^	green, fruit
3	Hexanal-M	C66251	1092.5	454.779	6.16 ± 0.14 ^b^	6.89 ± 0.15 ^a^	6.68 ± 0.14 ^ab^	6.66 ± 0.31 ^ab^	6.82 ± 0.56 ^a^	fruity, fatty, green, grassy
4	Hexanal-D	C66251	1091.7	453.82	8.05 ± 0.24 ^a^	2.06 ± 0.21 ^b^	1.77 ± 0.06 ^c^	1.49 ± 0.12 ^c^	1.63 ± 0.17 ^c^	fruity, fatty, green, grassy
5	3-Methylbutanal	C590863	848.3	259.115	1.52 ± 0.03 ^a^	0.81 ± 0.02 ^c^	0.79 ± 0.04 ^c^	0.85 ± 0.04 ^c^	1.09 ± 0.05 ^b^	sweet, fruity, chocolate-like
6	nonanal-M	C124196	1395.8	1320.484	1.79 ± 0.07 ^d^	9.26 ± 0.58 ^a^	8.82 ± 0.17 ^a^	8.20 ± 0.18 ^b^	7.36 ± 0.25 ^c^	orange, rose
7	heptanal-M	C111717	1189.5	668.819	0.49 ± 0.02 ^c^	3.05 ± 0.23 ^a^	2.91 ± 0.06 ^a^	2.54 ± 0.15 ^b^	2.55 ± 0.15 ^b^	fruity, fresh, green, sweet, herbal
8	Butyraldehyde-D	C123728	845.7	257.622	1.71 ± 0.02 ^a^	0.33 ± 0.02 ^c^	0.33 ± 0.01 ^c^	0.32 ± 0.02 ^c^	0.44 ± 0.03 ^b^	pungent
9	Butyraldehyde-M	C123728	847.3	258.556	2.92 ± 0.14 ^e^	4.98 ± 0.17 ^d^	5.46 ± 0.11 ^c^	6.42 ± 0.04 ^b^	8.15 ± 0.21 ^a^	pungent
10	octanal-M	C124130	1294.6	1016.887	0.96 ± 0.04 ^c^	6.22 ± 0.77 ^a^	6.15 ± 0.35 ^a^	5.55 ± 0.46 ^ab^	5.12 ± 0.44 ^b^	fatty, orange
11	octanal-D	C124130	1293.4	1013.09	0.29 ± 0.01 ^b^	1.09 ± 0.12 ^a^	1.17 ± 0.11 ^a^	1.19 ± 0.06 ^a^	1.20 ± 0.05 ^a^	fatty, orange
12	(E, E)-2,4-heptadienal	C4313035	1023	374.873	3.40 ± 0.16 ^a^	2.23 ± 0.11 ^b^	2.21 ± 0.06 ^b^	2.21 ± 0.10 ^b^	2.29 ± 0.19 ^b^	fatty, green
13	5-Methylfurfural	C620020	932.4	307.316	8.85 ± 0.33 ^e^	21.50 ± 0.88 ^b^	22.40 ± 0.22 ^a^	20.19 ± 0.58 ^c^	14.66 ± 0.69 ^d^	NA
14	ethyl 3-phenylpropanoate	C2021285	1362.2	1219.73	0.67 ± 0.07 ^e^	1.05 ± 0.03 ^d^	1.26 ± 0.09 ^c^	1.42 ± 0.10 ^b^	1.73 ± 0.07 ^a^	fruit, honey
15	isovaleric acid, methyl ester-M	C556241	1012.4	362.634	5.49 ± 0.15 ^a^	4.96 ± 0.09 ^b^	4.22 ± 0.10 ^c^	3.34 ± 0.09 ^d^	2.96 ± 0.15 ^e^	herbal, fruit
16	isovaleric acid, methyl ester-D	C556241	1015.1	365.734	11.27 ± 0.36 ^a^	1.48 ± 0.09 ^b^	1.14 ± 0.08 ^b^	1.15 ± 0.04 ^b^	1.19 ± 0.20 ^b^	herbal, fruit
17	Hexyl acetate-M	C142927	989.6	340.149	0.14 ± 0.01 ^c^	0.25 ± 0.03 ^ab^	0.21 ± 0.02 ^b^	0.25 ± 0.02 ^ab^	0.31 ± 0.08 ^a^	pleasant fruity, apple, cherry, pear, floral
18	Hexyl acetate-D	C142927	990.9	340.879	0.16 ± 0.01 ^b^	1.26 ± 0.11 ^a^	1.02 ± 0.09 ^a^	1.12 ± 0.13 ^a^	1.55 ± 0.60 ^a^	pleasant fruity, apple, cherry, pear, floral
19	Butyl acetate	C123864	854.2	262.483	0.14 ± 0.01 ^a^	0.06 ± 0.02 ^c^	0.07 ± 0.01 ^bc^	0.08 ± 0.01 ^bc^	0.09 ± 0.02 ^b^	fruity, sweet, pineapple
20	Ethyl lactate	C97643	835.7	251.898	0.31 ± 0.04 ^b^	0.45 ± 0.02 ^a^	0.41 ± 0.01 ^a^	0.41 ± 0.02 ^a^	0.46 ± 0.04 ^a^	rum, fruit, cream
21	Acetic acid, ethyl ester	C141786	891.2	283.73	0.01 ± 0.00 ^d^	0.11 ± 0.01 ^d^	0.45 ± 0.08 ^c^	0.61 ± 0.08 ^c^	0.86 ± 0.19 ^a^	pleasant, sweet, fruity
22	Amyl acetate	C628637	929.4	305.6	0.09 ± 0.03 ^b^	0.26 ± 0.08 ^a^	0.29 ± 0.04 ^a^	0.27 ± 0.04 ^a^	0.29 ± 0.03 ^a^	sweet, banana-like
23	1-Penten-3-one	C1629589	1026.6	378.94	0.46 ± 0.03 ^d^	0.64 ± 0.01 ^c^	0.68 ± 0.02 ^c^	0.79 ± 0.03 ^b^	1.00 ± 0.06 ^a^	pungent
24	2,3-Butanedione	C431038	985.8	337.958	0.10 ± 0.02 ^d^	0.24 ± 0.01 ^c^	0.22 ± 0.01 ^c^	0.29 ± 0.01 ^b^	0.34 ± 0.02 ^a^	quinone
25	2-Butanone	C78933	868.2	270.497	2.25 ± 0.11 ^a^	0.57 ± 0.02 ^d^	0.62 ± 0.03 ^d^	0.76 ± 0.02 ^c^	0.95 ± 0.03 ^b^	NA
26	Methyl heptenone	C110930	1339.5	1151.847	0.26 ± 0.02 ^c^	0.81 ± 0.06 ^ab^	0.80 ± 0.08 ^b^	0.86 ± 0.04 ^ab^	0.92 ± 0.07 ^a^	fruity, lemon
27	2-Methyltetrahydrofuran-3-one	C3188009	1290.5	1003.458	0.25 ± 0.01 ^e^	1.05 ± 0.06 ^d^	1.15 ± 0.02 ^c^	1.28 ± 0.05 ^b^	1.48 ± 0.05 ^a^	wintergreen
28	3-Octanone	C106683	1013.7	364.123	0.13 ± 0.03 ^b^	0.17 ± 0.02 ^a^	0.11 ± 0.01 ^b^	0.09 ± 0.01 ^c^	0.07 ± 0.01 ^c^	fruity, lavender
29	Acetic acid	C64197	1506.8	1653.411	3.60 ± 0.23 ^e^	9.12 ± 0.44 ^d^	10.06 ± 0.17 ^c^	11.45 ± 0.47 ^b^	13.21 ± 0.53 ^a^	sourness, pungent
30	pentanoic acid	C109524	915.5	297.651	1.42 ± 0.10 ^a^	0.77 ± 0.12 ^b^	0.70 ± 0.05 ^bc^	0.58 ± 0.06 ^c^	0.56 ± 0.07 ^c^	unpleasant
31	Linalool oxide-M	C60047178	1054.2	410.658	0.73 ± 0.04 ^a^	0.59 ± 0.09 ^a^	0.68 ± 0.13 ^a^	0.59 ± 0.05 ^a^	0.69 ± 0.12 ^a^	floral, woody-earthy
32	3-Methylpentanol	C589355	848.2	259.029	0.09 ± 0.00 ^c^	0.25 ± 0.03 ^b^	0.28 ± 0.04 ^ab^	0.27 ± 0.02 ^ab^	0.31 ± 0.04 ^a^	fruity, green, slightly pungent
33	Ethanol	C64175	890.2	283.158	0.30 ± 0.01 ^c^	0.47 ± 0.02 ^a^	0.35 ± 0.05 ^b^	0.42 ± 0.03 ^a^	0.42 ± 0.03 ^a^	alcohol
34	4-Ethylphenol	C123079	1206.1	721.533	1.04 ± 0.05 ^a^	0.57 ± 0.03 ^c^	0.61 ± 0.02 ^c^	0.70 ± 0.05 ^b^	0.73 ± 0.06 ^b^	woody-phenolic, sweet
35	4-Methylguaiacol	C93516	1144.1	566.278	0.12 ± 0.01 ^d^	0.44 ± 0.02 ^c^	0.53 ± 0.02 ^b^	0.70 ± 0.02 ^a^	0.68 ± 0.02 ^a^	sweet, spicy, vanilla-like
36	2,6-Dimethylpyrazine	C108509	887.3	281.479	0.29 ± 0.01 ^d^	0.85 ± 0.03 ^c^	0.94 ± 0.05 ^b^	1.15 ± 0.03 ^a^	1.16 ± 0.05 ^a^	coffee, fried peanuts
37	Methylpyrazine	C109080	836.3	252.24	1.06 ± 0.08 ^d^	1.58 ± 0.04 ^c^	1.59 ± 0.04 ^c^	1.73 ± 0.04 ^b^	1.82 ± 0.05 ^a^	nutty, cocoa, green, roasted, chocolate, meaty
38	Pyrazine, 2-ethyl-6-methyl-	C13925036	991	340.956	0.55 ± 0.04 ^d^	2.79 ± 0.11 ^c^	3.02 ± 0.13 ^c^	3.62 ± 0.09 ^b^	4.30 ± 0.47 ^a^	roasted baked potato
39	2-Ethylfuran	C3208160	990.4	340.636	0.30 ± 0.01 ^d^	0.85 ± 0.02 ^a^	0.68 ± 0.02 ^b^	0.58 ± 0.02 ^c^	0.55 ± 0.08 ^c^	burnt, coffee
40	2-Acetylpyrrole	C1072839	1113.5	497.191	0.10 ± 0.01 ^a^	0.07 ± 0.00 ^c^	0.07 ± 0.01 ^c^	0.08 ± 0.01 ^bc^	0.09 ± 0.02 ^ab^	bread, walnut, licorice
41	Limonene	C138863	1146.9	572.615	0.06 ± 0.00 ^b^	0.46 ± 0.03 ^a^	0.47 ± 0.07 ^a^	0.46 ± 0.05 ^a^	0.40 ± 0.04 ^a^	pleasant, pine-like, lemon-like
42	Dimethyl disulphide	C624920	1122.8	518.217	0.14 ± 0.01 ^a^	0.13 ± 0.01 ^a^	0.13 ± 0.01 ^a^	0.14 ± 0.01 ^a^	0.14 ± 0.01 ^a^	NA
43	o-Xylene	C95476	907.1	292.82	0.24 ± 0.04 ^c^	0.44 ± 0.09 ^b^	0.46 ± 0.07 ^b^	0.68 ± 0.06 ^a^	0.78 ± 0.13 ^a^	sweet
44	2,2,4,6,6-Pentamethylheptane-D	C13475826	921.2	300.897	2.68 ± 0.15 ^a^	0.60 ± 0.05 ^bc^	0.66 ± 0.07 ^bc^	0.56 ± 0.06 ^c^	0.74 ± 0.04 ^b^	NA
45	2,2,4,6,6-Pentamethylheptane-M	C13475826	922	301.379	0.09 ± 0.02 ^b^	0.08 ± 0.01 ^bc^	0.07 ± 0.00 ^c^	0.08 ± 0.00 ^bc^	0.11 ± 0.01 ^a^	NA
46	N-nitroso-N-methylaniline	C614006	1305	1048.326	0.09 ± 0.00 ^c^	0.59 ± 0.07 ^a^	0.49 ± 0.05 ^b^	0.53 ± 0.02 ^ab^	0.50 ± 0.05 ^b^	NA
47	N, N-Dimethylformamide	C68122	1311	1066.309	0.20 ± 0.05 ^c^	0.69 ± 0.09 ^a^	0.59 ± 0.02 ^b^	0.57 ± 0.05 ^b^	0.59 ± 0.03 ^b^	ammonia-like

Note: Odor description is taken from the ChemicalBook database. M—monomer; D—dimer; RI—retention index; Rt—retention time; NA—no aroma description. Different lowercase letters denoted significant difference (*p* < 0.05).

## Data Availability

The data presented in this study are available on request from the corresponding author. The data are not publicly available due to privacy restrictions.
